# A clinical case of insulin autoimmune syndrome with monoclonal gammopathy of uncertain significance; complexity in management

**DOI:** 10.1093/omcr/omae054

**Published:** 2024-06-07

**Authors:** Liam Clifford, Flavian Joseph, Tripti Joshi

**Affiliations:** Department of Endocrinology, Gosford Hospital, Gosford, NSW, Australia; Department of Endocrinology, Gosford Hospital, Gosford, NSW, Australia; University of Newcastle, Newcastle, NSW, Australia; Department of Endocrinology, Gosford Hospital, Gosford, NSW, Australia; University of Newcastle, Newcastle, NSW, Australia

**Keywords:** insulin autoimmune syndrome, insulin, C-peptide, hypoglycaemia, anti-insulin antibodies, MGUS

## Abstract

Insulin autoimmune syndrome (IAS) is a rare cause of spontaneous hypoglycaemia. We discuss a 91-year-old Caucasian lady who presented with syncope and episodic adrenergic and neuroglycopenic symptoms. Despite significantly elevated insulin, C-peptide, and proinsulin levels with the presence of anti-insulin antibodies, a pancreatic mass was not identified. Serum immunoelectrophoresis demonstrated monoclonal gammopathy of undetermined significance (MGUS). Treatment involved high-dose steroids, diazoxide, corn starch and acarbose, however the patient passed away four months later due to worsening co-morbidities. The management of IAS in the setting of MGUS is challenging.

## Introduction

Insulin autoimmune syndrome (IAS) was first identified in 1970, predominantly in the East Asian population [1]. IAS is characterised by hyperinsulinaemic hypoglycaemia, an elevated insulin autoantibody (IAA) titre, and absence of pancreatic islet cell dysfunction or exogenous insulin [2]. Aetiologies include exposure to sulfhydryl compounds, viral infections, autoimmune disease, and haematological malignancies such as monoclonal gammopathy of undetermined significance (MGUS) and multiple myeloma [3]. The Caucasian population has been linked more frequently with autoimmune and haematological malignancy-associated IAS [2]. It is estimated the incidence of IAS is 0.017 per 100 000, and is most common in women in the fourth decade of life [4]. This is the first reported case of MGUS-associated IAS in Australia.

## Case report

A 91-year-old Caucasian lady presented with syncope. Initial blood glucose level was 2.2 mmol/l with confusion, which resolved with short-acting carbohydrates. No seizure activity. For the last year she had experienced multiple episodes of confusion with tremors, resolving after eating. Past medical history included hypothyroidism following a thyroidectomy (negative thyroid autoantibodies), heart failure with preserved ejection fraction, obstructive sleep apnoea, nephrectomy for hypertension with chronic kidney disease, transient ischaemic attack, hypercholesterolaemia, hypertension, and osteoarthritis. No history of diabetes mellitus, use of agents for diabetes, or prior exposure to compounds containing sulfhydryl groups. Family history was significant for a son who had neonatal hypoglycaemia. Medications included aspirin, atorvastatin, perindopril, spironolactone, verapamil, moxonidine, rabeprazole, levothyroxine, cholecalciferol, pregabalin, tramadol, and paracetamol. She required assistance with activities of daily living and mobility. Minimal alcohol intake and never smoked.

On admission she was haemodynamically stable and afebrile. Cardiogenic causes of syncope were excluded. She experienced multiple hypoglycaemic events several hours after carbohydrates (Table 1). Biochemistry revealed an estimated glomerular filtration rate of 45 ml/min/1.73 m2 (reference range (RR) >60), thyroid stimulating hormone level 0.56mIU/l (RR 0.4–5.0) and no anaemia. Morning cortisol serum level 333 nmol/l (RR 100–535), insulin growth factor-1 level 138ug/l (RR 54–204), growth hormone level 1.4mIU/(RR <8), and a glycated haemoglobin A1 level of 36 mmol/mol (RR <48).

**Table 1:** Spontaneous Hypoglycaemia in a Post-Prandial Pattern

Time (24 h)	Capillary glucose (mmol/l)	Intervention
2:45	6.8	
6:01	4.6	
8:17	6.9	
11:24	4.8	
14:42	8	
17:17	5.1	
21:00	3.3	Oral glucose
22:15	6.1	
23:52	6.9	
2:00	2.7	Oral glucose
2:25	5.1	
2:58	9.5	
6:06	1.6	Oral glucose
6:25	3.7	
6:42	7.3	
7:50	16.4	

**Figure 1:**
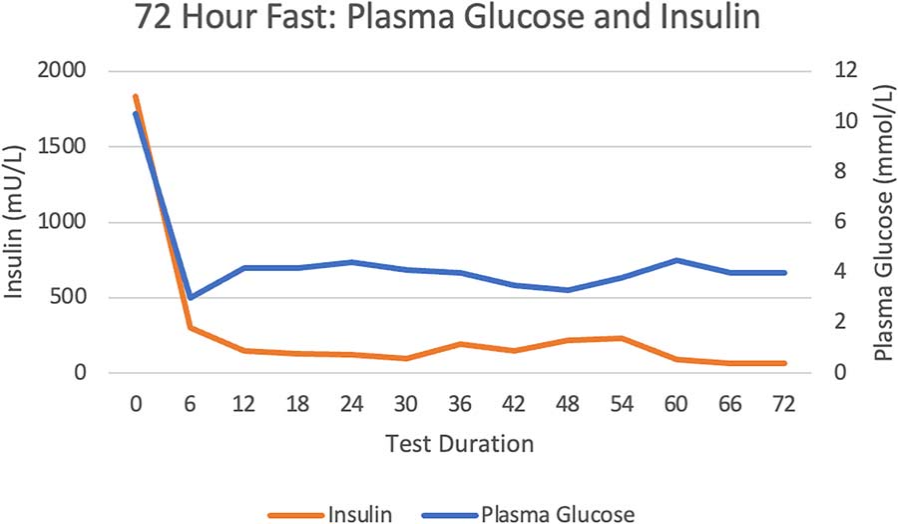
72-Hour Fast: Plasma Glucose and Insulin Levels.

**Figure 2:**
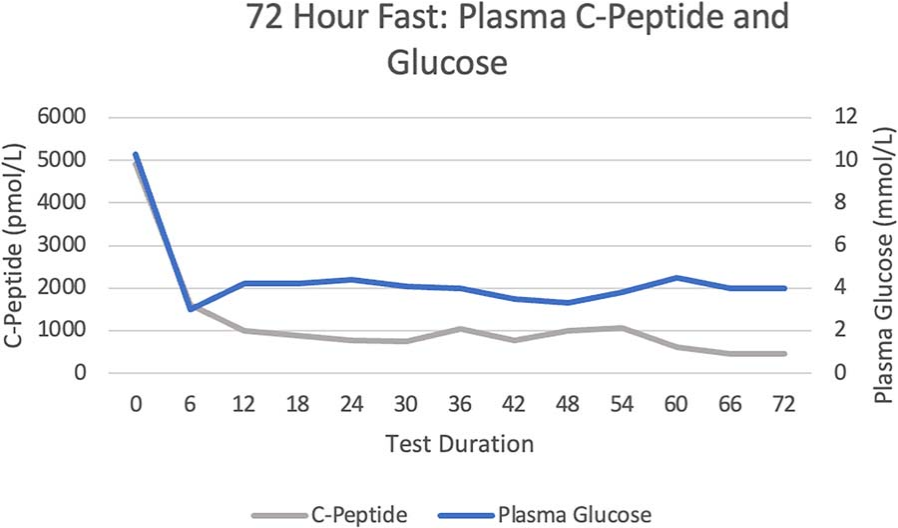
72 Hour Fast: Plasma C-Peptide and Glucose Levels.

**Figure 3:**
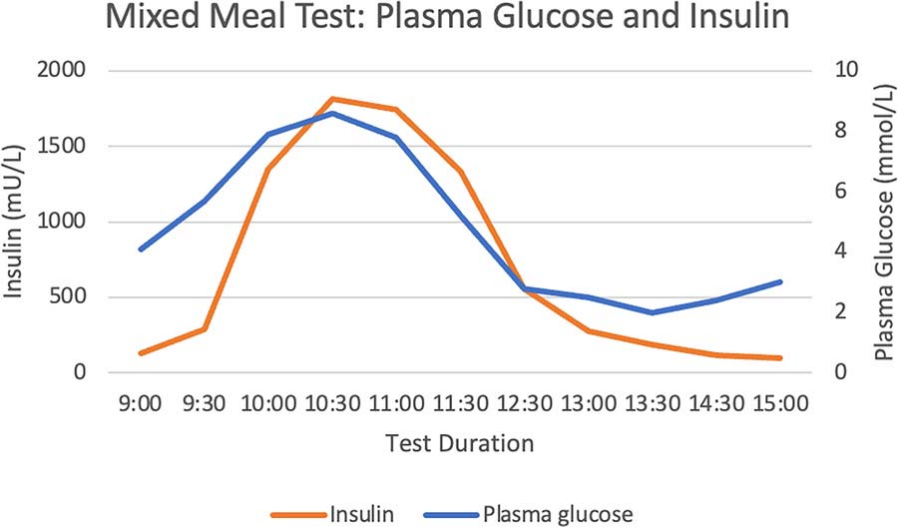
Mixed Meal Test: Plasma Glucose and Insulin Levels.

**Figure 4:**
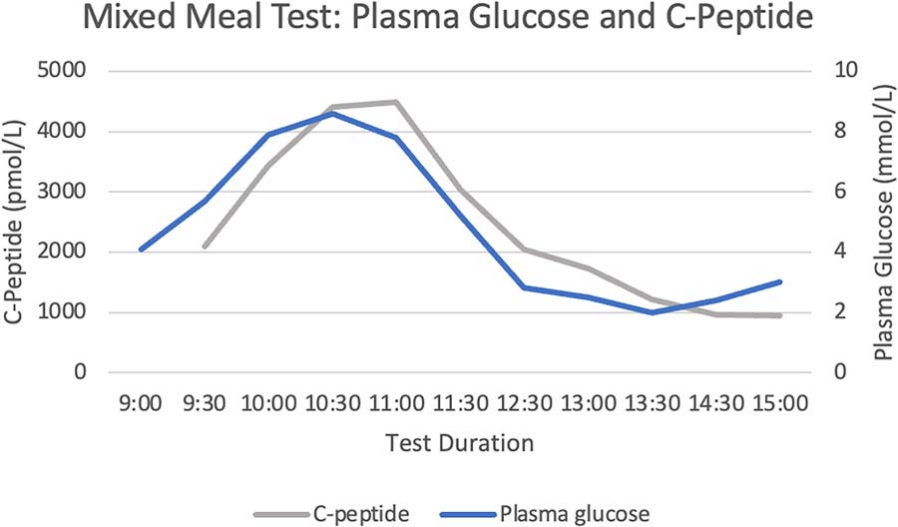
Mixed Meal Test: Plasma C-Peptide and Glucose Levels.

A 72-hour fast did not provoke significant hypoglycaemia or symptoms of neuroglycopenia (Figs 1 and 2). There were inappropriately elevated levels of insulin, proinsulin, and C-peptide whilst fasting, with inappropriately normal β-hydroxybutyrate levels. Sulfonylurea screening was negative. IAA titre 46.7kIU/l (RR <0.4). A mixed meal test demonstrated a nadir of hypoglycaemia at 2 mmol/l at 4.5 h, with a corresponding insulin level 187.1 mU/l (RR <10) and C-peptide level 1221 pmol/l (RR 260–1730) (Figs 3 and 4). Cessation of the test demonstrated a proinsulin level >100 pmol/l (RR <13.3) and IAA titre >50kIU/l.

Whole body contrast-enhanced computer tomography (CT) demonstrated no overt abnormalities or lymphadenopathy. A Gallium68 DOTATE positron emission tomography scan showed no pancreatic mass or increased activity. A monoclonal band was detected in the gamma region with a paraprotein quantified at 4 g/l (RR <0) however serum and urine free light chains as well as the electrophoresis and immunophoresis were negative. A CT skeletal survey was negative, indicating MGUS.

Treatment initially consisted of oral glucose and dextrose infusions. Diet was modified to low glycaemic indices. Prednisolone was uptitrated to 60 mg due to persisting hypoglycaemia, and slowly weaned down to 45 mg (threshold which prevented hypoglycaemia). Corn starch was mixed with daily milk to reduce glycaemic excursions. Active treatment of MGUS was not pursued following discussion with Haematology. Following discharge, the patient re-presented with worsening heart failure due to steroids. A repeat IAA titre was 31.2kIU.L. Acarbose then diazoxide were introduced with improvement, however the admission was complicated by pulmonary embolism and an upper gastrointestinal bleed, and the patient passed away.

## Discussion

In the Caucasian population, IAS is rare. Comparatively, an insulinoma is the most common form of endogenous hyperinsulinaemic hypoglycaemia at 0.16 per 100 000 [4]. Human Leukocyte Antigens (HLA) are associated with the disease, specifically HLA-DRB1^*^046, DRB1^*^0403, and DRB1^*^0407. HLA-DRB1^*^046 may account for the higher prevalence seen in Japan and other East Asian populations [5].

Two triggers have been identified: viral infections and exposure to sulfhydryl compounds, most commonly methimazole, and more recently, alpha lipoic acid [6]. These compounds render native insulin more immunogenic by cleaving the disulphide bonds linking peptide chains A and B [7]. Viruses like measles, mumps, and varicella zoster act as superantigens, enhancing the immunogenicity of the insulin molecule [8]. There are several case reports describing the association of MGUS and myeloma with IAS [9].

Hypoglycaemia secondary to IAA is due to binding of secreted insulin directly, reducing the activity and prolonging the half-life. This produces insufficient control of the glycaemic excursions, and compensatory over-secretion of insulin. After euglycaemia is achieved, a large quantity of insulin remains in the bloodstream, leading to hypoglycaemia [2]. This is likely why we were unable to provoke hypoglycaemia with a 72-hour fast however late reactive hypoglycaemia was demonstrated 240 min after ingesting mixed foods.

For MGUS, it is postulated that the production of a monoclonal antibody binds to both the insulin molecule and receptor. These antibodies have a higher affinity to the insulin receptor and cause dissociation from the immune complex, with more antibodies available to bind to the insulin receptor resulting in hypoglycaemia [9]. Spontaneous remission has been observed in patients where no cause was identified [5].

There are no established management guidelines for IAS-associated MGUS. The use of small meals with low glycaemic indices is recommended, and administration of short-acting carbohydrates in the event of a hypoglycaemic episode. If the episodes are severe/refractory, the use of high-dose glucocorticoids, steroid-sparing agents (rituximab, azathioprine) [4, 10], or plasmapheresis may be required to decrease the anti-insulin-binding monoclonal antibody [9]. The combination of IAS and MGUS requires further evaluation.

## Conclusion

MGUS-associated IAS is challenging to treat as production of antibodies may not remit whilst the underlying condition is active. Patients may require lifelong dietary modifications with steroid therapy. Additional measures include corn starch, acarbose and diazoxide. Plasmapheresis can be considered to reduce the degree of circulating antibodies if avoidance of hypoglycaemia is not achieved.
